# Supplementary material for: Effectiveness of Hyperthermic Intraperitoneal Chemotherapy Associated with Cytoreductive Surgery in the Treatment of Advanced Ovarian Cancer: Systematic Review and Meta-Analysis
Source: J Pers Med. 2023 Jan 30;13(2):258. doi: 10.3390/jpm13020258 (PMC9960788; doi:10.3390/jpm13020258)
Supplement: Supplementary file 1 [file jpm-13-00258-s001.zip › jpm-1979283-supplementary.pdf]

Suplementary material

Table S1:Histological types of the selected studies

	Arm	Serous n(%)	Endometrioide n(%)	Clear cels n(%)	Mucinous n(%)	Others n(%)
Cheol Lim M. et al. [17]	Control	38(88,4)	2 (4,7)	1 (2,3)	0	2 (4,7)
	Experimental	32(94,1)	0	0	0	2 (5,9)
Van Driel W.J. et al. [16]	Control	109 (88,6)	1 (0,8)	5 (4,1)	2 (1,6)	6 (4,9)
	Experimental	116 (95)	3 (2,5)	0	1 (0,8)	2 (1,6)
Ceresoli M. et al. [23]	Control	47 (95,9)	0	1 (2)	0	0
	Experimental	25 (89,3)	3 (10,7)	0	0	0
Mendevil A.A. et al. [24]	Control	45 (65,2)	5 (7,3)	6 (8,7)	3 (4,3)	10 (13)
	Experimental	48 (69,6)	4 (5,8)	4 (5,8)	4 (5,8)	9 (14,6)

N: population

Figure S1: Bias Risk Analysis

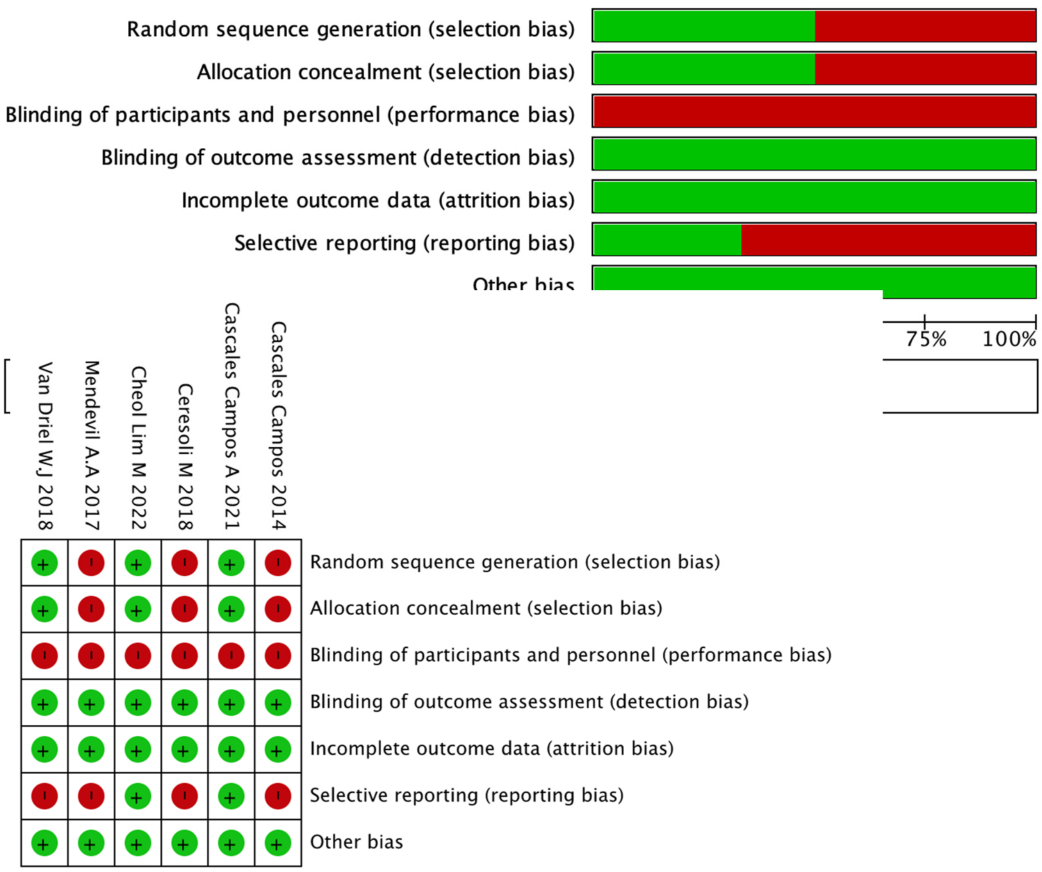

Figure S1: Bias Risk Analysis; (A) Risk of bias graph: Author's assessment of each bias presented as a percentage. (B) Summary Risk of bias: Author's assessment of each possible bias of the included studies.

Table S2. Main results of the included articles.

	Arm	DFS (Median Months)	HR	IC 95%	*p*	OS (Median Months)	HR	IC 95%	*p*
Cheol Lim M. et al. [17]	Control	15.4	0.60	0.37–0.97	0.04	48.2	0.53	0.29–0.96	0.04
	Experimental	17.4				61.8			
Cascales Campos P. et al. [18]	Control	12	0.12	0.02–0.89	0.038	45	0.05	0.0031–0.8	0.035
	Experimental	18				52			
Van Driel W.J. et al. [16]	Control	10.7	0.66	0.50–0.87	<0.01	33.9	0.67	0.48–0.94	0.02
	Experimental	14.2				45.7			
Ceresoli M. et al. [23]	Control	13.23	1.41	0.52–3.83	0.454	32.53	0.34	0.12–0.99	0.048
	Experimental	13.96				43			
Mendevil A.A. et al. [24]	Control	20.0	2.10	1.29–3.42	<0.01	33.6	1.22	0.64–2.67	0.95
	Experimental	25.1				33.8			
Cascales Campos P. et al. [25]	Control	26.7	0.37	0.21–0.68	<0.01	NE	NE	NE	NE
	Experimental	39.1				NE	NE	NE	NE

NE, Not specified; DFS, Disease free time/disease free survival; OS, Overall survival/overall survival; HR, Hazard ratio; CI, Confidence interval; *p*, *p* value.

Table S3: Grade III-V complications in the first postoperative month.

	Arm	Major complications (grade 3-5 complications)during first postoperative month (n) (%)	Relative Risk (RR)	IC 95%	P
Cheol Lim M. et al. [17]	Control	80 (87%)	1.07	0.98-1.18	0.14
	Experimental	86 (93,5%)			
Cascales Campos P. et al. [18]	Control	10 (27,8%)	1.03	0.49-2.16	0.94
	Experimental	10 (28,6%)			
Van Driel W.J. et al. [16]	Control	30 (25%)	0.95	0.61-1.45	0.79
	Experimental	32 (27%)			
Ceresoli M. et al. [23]	Control	5 (17,9%)	1.40	0.50-3.89	0.52
	Experimental	7 (25%)			
Mendevil A.A. et al. [24]	Control	NE	NE	NE	NE
	Experimental	NE	NE	NE	NE
Cascales Campos P. et al. [25]	Control	NE	NE	NE	NE
	Experimental	NE	NE	NE	NE

NE; Not especified, n; population.
